# Supplementary figures and images for: Evasion of host antioxidative response via disruption of NRF2 signaling in fatal Ehrlichia-induced liver injury
Source: PLoS Pathog. 2023 Nov 13;19(11):e1011791. doi: 10.1371/journal.ppat.1011791 (PMC10681308; doi:10.1371/journal.ppat.1011791)

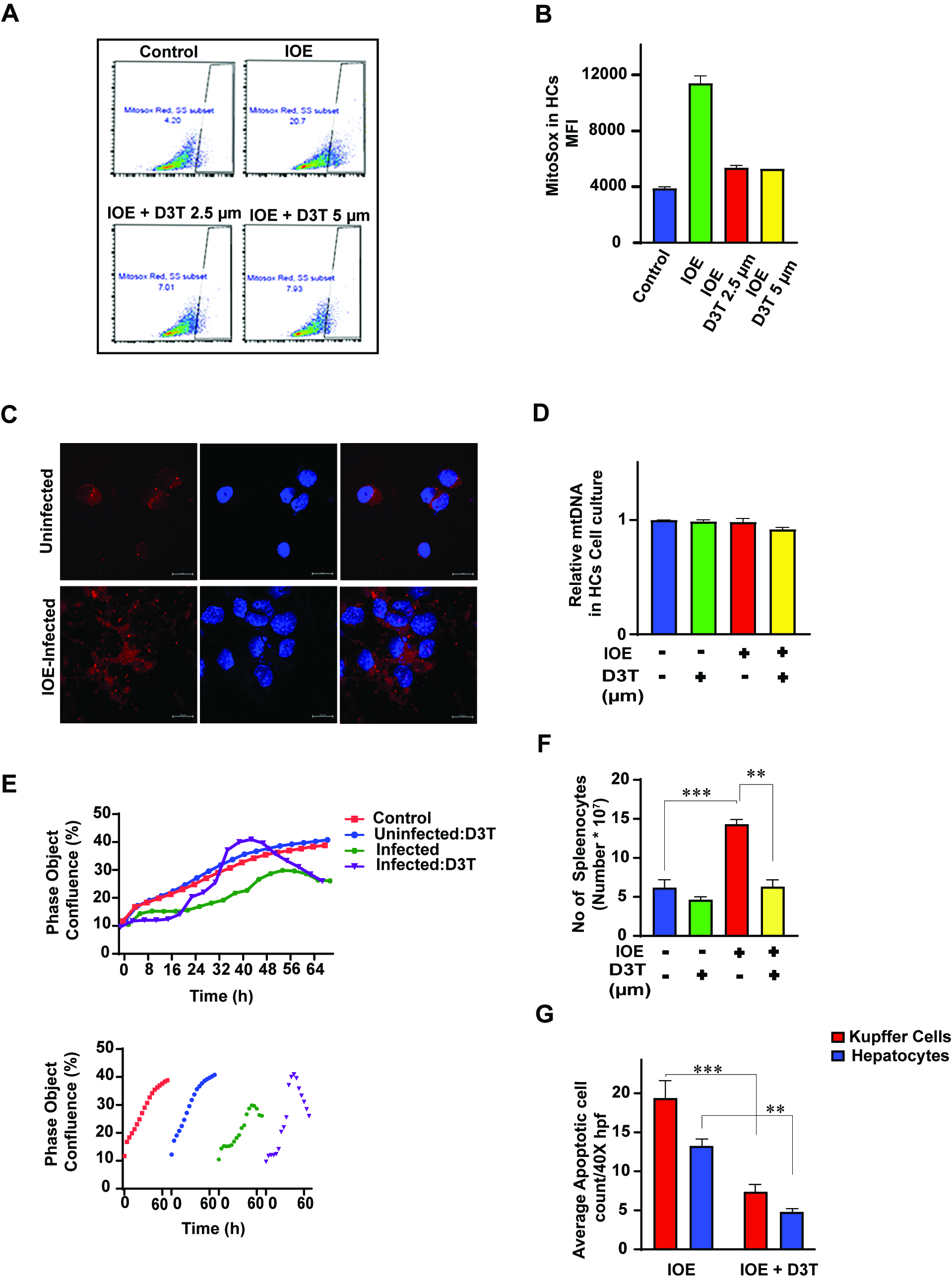

Supplement: S1 Fig — (A) Dot blot data analyzing intracellular expression of mitochondrial ROS in uninfected or infected HCs, cultured with or without D3T at 24hr p.i. using Mitosox Red. (B) Analysis of MFI of Mitosox Red using flow cytometry of same cells described in (A). (C) Immunofluorescence staining of uninfected and IOE-infected HCs and labeled with Mitotracker (Red) and DAPI (blue). Scale bar 20 μm. (D) Graph showing ratio of mitochondrial DNA and nuclear DNA measured by q-RT PCR. (E) Representative graph showing the cell proliferation assay results in uninfected, IOE-infected, and D3T-treated IOE-infected HCs. The assay measures the ability of these cells to proliferate or grow in number over 68h, and the graph shows how treatment with D3T affects the proliferation of IOE-infected HCs compared to uninfected or untreated IOE-infected or D3T treated-IOE infected cells. (F) Graph showing splenocytes in uninfected, D3T treated uninfected, IOE-infected, and D3T treated IOE-infected mice. (G) Quantification of TUNEL-positive kupffer cells and HCs/ 40x hpf in IOE-infected and D3T-treated-IOE-infected liver tissues on 7 day p.i. (TIF) [file ppat.1011791.s001.tif]
