# Supplementary material for: Evasion of host antioxidative response via disruption of NRF2 signaling in fatal Ehrlichia-induced liver injury
Source: PLoS Pathog. 2023 Nov 13;19(11):e1011791. doi: 10.1371/journal.ppat.1011791 (PMC10681308; doi:10.1371/journal.ppat.1011791)
Supplement: S1 Table — (DOCX) [file ppat.1011791.s002.docx]

| **Gene Categories**  **Table S1** | **Protein and gene symbols** | **Analyzed proteins/**  **genes** | **Name** | **Function** | **Uninfected** | **EM-Infected** | **IOE-Infected** |
| --- | --- | --- | --- | --- | --- | --- | --- |
| **Anti-oxidative**  **Response** | NRF2  *nrf2* | Protein  RNA | Nuclear factor erythroid 2-related factor 2 | Transcription factor (TF) that triggers the expression of antioxidant enzymes and detoxification molecules following its nuclear translocation. It plays a role in anti-oxidative response to maintain cellular homeostasis. NRF also plays a crucial role in inducing an anti-oxidative response against stress or infections as well as regulation of genes involved in lipid metabolism (18-20, 29). | **+**  **(**nuclear) | **+**  **(**nuclear) | **-**  **(**No nuclear localization) |
|  | GPX4  *gpx4* | Protein  RNA | Glutathione Peroxidase 4 | Antioxidant enzyme that catalyzes the reduction of hydrogen peroxide and lipid hydroperoxides using glutathione as a reducing agent (21,22). | **+** | **+** | **-** |
|  | GPX3  *gpx4* | RNA | Glutathione Peroxidase 3 | Plasma glutathione peroxidase. It prevents the accumulation of H2O2 in the extracellular space. It plays a role in preventing oxidative stress and maintenance of redox balance (23,24). | **+** | **+** | **+** |
|  | TXNRD1  *txnrd1* | RNA | Thioredoxin Reductase 1 | An enzyme that belongs to the family of thioredoxin reductases family. It detoxifies bacterial toxins, electrophilic compounds, environmental toxins, and reactive intermediates(25). | **+** | **+** | **+++** |
|  | NQO1  *nqo1* | RNA | NAD(P)H: quinone oxidoreductase 1 | An enzyme that catalyzes the reduction of quinones and other electrophilic compounds involved in oxidative damage and diminishes their adverse effects on cellular components, including DNA, protein, and lipids(26-28). | **+** | **+** | **---** |
| **Mitochondrial gene** | PINK1  *pink1* | Protein  RNA | Phosphatase and tensin homolog (PTEN) Induced Putative Kinase | A protein kinase that plays a crucial role in maintaining mitochondrial health. It eliminates damaged or dysfunctional mitochondria via mitophagy(35-38). | **+** | **+** | **---** |
|  | PARKIN  *Park2* | Protein  RNA | Parkin RBR E3 ubiquitin protein ligase | E3 ubiquitin ligase. It gets recruited to the surface of the damaged mitochondria and tags them with ubiquitin to be removed via mitophagy(35-38). | **+** | **+** | **---** |
|  | ERP44  *erp44* | RNA | Endoplasmic reticulum protein 44 | A pH-regulated chaperone of the secretory pathway. It plays a role in protein quality control at the ER-Golgi interface(40,41). | **+** | **+** | **---** |
| **Unfolded protein response** | PERK  *Perk* | RNA | Protein kinase R-like endoplasmic reticulum kinase | It initiates UPR upon ER stress. PERK phosphorylates eukaryotic translation initiation factor 2 alpha (eIF2α), leading to a global decrease in protein synthesis. PERK activation also leads to the upregulation of ER protein folding and ER-associated degradation (ERAD) proteins(42,43). | **+** | **+** | **+++** |
|  | IRE1α  *ire1α* | RNA | Inositol-requiring enzyme 1 | It has both endoribonuclease and kinase activity. IRE1 endonuclease activity leads to splicing introns from mRNA encoded mRNA encoding X-box binding protein 1 (XBP1). The spliced XBP1 then regulates genes involved in ER function and protein folding. IRE1 activates c-Jun N-terminal kinase (JNK) and the NF-κB pathways with its kinase activity(42, 43). | **+** | **++** | **+++** |
|  | ATF6  *atf6* | RNA | Activating transcription factor 6 | A transcription factor that binds to ER stress response elements (ERSEs) in the promoter regions of target genes responsible for ER homeostasis and alleviating ER stress(42,43). | **+** | **+** | **+++** |
|  | XBP1  *xbp1* | RNA | X-box binding protein 1 | It is a downstream target of the IRE1 UPR branch. It is an unspliced form of XBP1 (XBP1u) (42,43). | **+** | **++** | **+++** |
|  | XBP1s  *xbp1s* | RNA | Spliced form of X-box binding protein 1 | A transcriptionally active form of XBP1 that regulates genes involved in ER function, protein folding, and ER-associated degradation (ERAD) pathway(42,43). | **+** | **++** | **+++** |
|  | CHOP  *chop* | RNA | C/EBP homologous protein | A CCAAT/enhancer binding protein that regulates genes involved in proliferation, differentiation, and energy metabolism. It regulates many anti-apo- and pro-apoptotic proteins (44, 45). | **+** | **+** | **+++** |
| **ER-associated protein degradation** | EDEM1  *edem1* | RNA | ER Degradation Enhancing Alpha-Mannosidase-Like Protein 1 | It is a vital protein for quality control in the ER. It helps in the disposal and degradation of misfolded proteins through ER-associated degradation(46). | **+** | **+** | **+++** |
|  | GADD34  *gadd34* | RNA | Growth arrest and DNA damage-inducible protein 34 | It dephosphorylates α subunit of eukaryotic translation initiation factor 2 (eIF-2α) to inhibit protein synthesis and attenuate ER stress(47). | **+** | **+** | **+++** |
|  | DR5  *dr5* | RNA | Death receptor 5 | A TNF-receptor superfamily that mediates apopotic cell death (48). | **+** | **+** | **+++** |
