# Supplementary material for: Evasion of host antioxidative response via disruption of NRF2 signaling in fatal Ehrlichia-induced liver injury
Source: PLoS Pathog. 2023 Nov 13;19(11):e1011791. doi: 10.1371/journal.ppat.1011791 (PMC10681308; doi:10.1371/journal.ppat.1011791)
Supplement: S2 Table — (DOCX) [file ppat.1011791.s003.docx]

**S2 Table**

|  | **Gene Name** | **Primer** | **5'-------------------3'** |
| --- | --- | --- | --- |
| **1** | *gpx4* | FWD | GCCTGGATAAGTACAGGGGTT |
|  | *gpx4* | REV | CATGCAGATCGACTAGCTGAG |
| **2** | *gpx3* | FWD | CCTTTTAAGCAGTATGCAGGCA |
|  | *gpx3* | REV | GGGGAGTATCTCCGAGTTCTC |
| **3** | *txnrd1* | FWD | GGGTCCTATGACTTCGACCTG |
|  | *txnrd1* | REV | AGTCGGTGTGACAAAATCCAAG |
| **4** | *nqo1* | FWD | AGGATGGGAGGTACTCGAATC |
|  | *nqo1* | REV | AGGCGTCCTTCCTTATATGCTA |
| **5** | *nrf2* | FWD | TCTCCTCGCTGGAAAAAGAA |
|  | *nrf2* | REV | AATGTGCTGGCTGTGCTTTA |
| **6** | *dsb* | FWD | CAGGATGGTAAAGTACGTGTGA |
|  | *dsb* | REV | TAGCTAACGCTGCCTGAACA |
| **7** | *gapdh* | FWD | CAACTACATGGTCTACATGTTC |
|  | *gapdh* | REV | TCGCTCCTGGAAGATG |
| **8** | *pink1* | FWD | TTCTTCCGCCAGTCGGTAG |
|  | *pink1* | REV | CTGCTTCTCCTCGATCAGCC |
| **9** | *parkin* | FWD | GAGGTCGATTCTGACACCAGC |
|  | *parkin* | REV | CCGGCAAAATCACACGCAG |
| **10** | *erp44* | FWD | TGCGGTCTTCCTGTCTTTAGC |
|  | *erp44* | REV | AACGACACCAGTCAGCATAAAA |
| **11** | *perk* | FWD | ACTCCTGTCTTGGTTGGGTCTGAT |
|  | *perk* | REV | CGTGCTCCGATTCCTTTCT |
| **12** | *ire1α* | FWD | CCTTTGCTGATAGTCTCTGCCCAT |
|  | *ire1α* | REV | TTACCACCAGTCCATCGCCATT |
| **13** | *atf6* | FWD | CGTTCCTGAGGAGTTGGATTTG |
|  | *atf6* | REV | GCTTCTCTTCCTTCAGTGGCTCTA |
| **14** | *xbp1* | FWD | TGTCCATTCCCAAGCGTGTTCT |
|  | *xbp1* | REV | TGGAGCAGCAAGTGGATTT |
| **15** | *xbp1s* | FWD | TTACGGGAGAAAACTCACGGC |
|  | *xbp1s* | REV | GGGTCCAACTTGTCCAGAATGC |
| **16** | *chop* | FWD | TGAAAGCAGAACCTGGTCCA |
|  | *chop* | REV | CACTGTTCATGCTTGGTGCA |
| **17** | *edem1* | FWD | CGGCTATGACAACTACATGG |
|  | *edem1* | REV | GTTCAGATTGGAAGGGTCTC |
| **18** | *gadd34* | FWD | CAGAACATCAAGCCACGGAAGA |
|  | *gadd34* | REV | AAAGTTGTCTCAGGTCCTCCTTCC |
| **19** | *dr5* | FWD | AAGCCTTGCAGAGAGGTATTGAC |
|  | *dr5* | REV | GCAGTTAGAGCATGACTGGAGAT |
| **20** | *caspase11* | FWD | ACAATGCTGAACGCAGTGAC |
|  | *caspase11* | REV | CTGGTTCCTCCATTTCCAGA |
| **21** | *cytochrome b (mtdna)* | FWD | TTGGGTTGTTTGATCCTGTTTCG |
|  | *cytochrome b (mtdna)* | REV | CTTCGCTTTCCACTTCATCTTACC |
| **22** | *β-actin* | FWD | CAGGATGCCTCTCTTGCTCT |
