# Supplementary material for: Evasion of host antioxidative response via disruption of NRF2 signaling in fatal Ehrlichia-induced liver injury
Source: PLoS Pathog. 2023 Nov 13;19(11):e1011791. doi: 10.1371/journal.ppat.1011791 (PMC10681308; doi:10.1371/journal.ppat.1011791)
Supplement: S1 Text — (DOCX) [file ppat.1011791.s004.docx]

**Evasion of Host Antioxidative Response via Disruption of NRF2 Signaling in Fatal *Ehrlichia*-Induced Liver Injury**

Aditya Kumar Sharma^1^, Abdeljabar El Andaloussi^1^, Nahed Ismail^1*^

^1^ Department of Pathology, College of Medicine, University of Illinois at Chicago, Illinois, USA

**Table of content**

- **Material and Methods**
  - **Histopathology, terminal deoxynucleotidyl transferase dUTP nick end labeling (TUNEL) and Transmission Electron Microscope (TEM)**
  - **Nuclear and cytoplasmic fractionation**
  - **Western Blot**
  - **RNA isolation and quantitative RT-PCR**
  - **Bacterial burden determination**
  - **Mitochondria isolation, cardiolipin staining, Mitochondrial membrane potential assay.**
  - **DCFDA / H2DCFDA Cellular ROS Assay**
  - **Cell Viability Assay**
  - **IncyCyte Cell Proliferation assay**
  - **Confocal microscopy**
  - **Statistical Analysis**

**Material and Methods**

**Histopathology, terminal deoxynucleotidyl transferase dUTP nick end labeling (TUNEL) and Transmission Electron Microscope (TEM)**

At different time points, experimental mice were sacrificed, and liver tissues were harvested, fixed in 10 % neutral buffered solution of formalin. The liver tissues were next processed as described before and stained by Hematoxylin and eosin (H&E)(13). Apoptotic cells were measured by TUNEL staining. Images were captured using Leica Aperio AT2 (Leica Biosystems) at 40x magnification. For TEM, infected or uninfected HCs were fixed and processed for TEM and analyzed by JEM-1011 Transmission Microscope (JEOL Ltd.) as described before(13).

**Nuclear and cytoplasmic fractionation**

For *in vivo* data, 20 mg of the liver tissue was cut, washed with PBS, and dried. The liver tissue was then resuspended into 200 ul of ice-cold CER-I buffer and homogenized using a Dounce homogenizer. For *in vitro* culture, HCs were harvested and centrifuged at 500 g for 5 minutes to collect cells. For isolation of the nuclear and cytoplasmic proteins from liver lysates or HCs, we used NE-PER™ Nuclear and Cytoplasmic Extraction kits as recommended by the manufacturer (78833, Thermofisher). All steps were performed on ice. Briefly, cells were washed with PBS and centrifuged at 500 × g for 5 minutes to obtain pellets. The supernatant was discarded, and the cell pellet was resuspended in 100 µl of ice-cold CER-I. The pellet was vigorously vortexed and then incubated in the CER-I buffer for 10 minutes. Following this incubation, ice-cold CER-II was added to the samples, vortexed, incubated for 1 min, and centrifugation at 16,000 x g for 5 minutes. The cytoplasmic supernatant was collected to clean pre-chilled tube on ice. The pellet containing nuclei was resuspended in NER-I reagent, and the resuspended pellet was vortexed for 15 sec every 10 minutes for a total of 40 minutes. The nuclear fraction was then centrifuged at 16,000 x g for 10 minutes and the supernatant containing the nuclear extract was transferred to a clean pre-chilled tube. The cytosolic and nuclear extracts were stored at -80°C until needed.

**Western Blot**

Liver tissues and HCs were lysed in T-PER lysis buffer (78510, Thermofisher) with Halt protease inhibitors (78430, Thermofisher). Standard Western blot procedure was followed as described before(10,13). These extracts (20µg) of protein were subjected to analysis through 4-20% SDS-PAGE and subsequent Western blotting. For analysis of total protein lysates, primary antibodies used are polyclonal anti-NRF2 (A1244, abclonal), polyclonal anti-Caspase11 (Clone 17D9, Novus Biologicals), polyclonal anti-GPX4 (52455, cell signaling), polyclonal anti-PINK1 (ab23707, Abcam), and anti-PARKIN (4211, cell signaling) at dilution 1:1000. As secondary antibody anti-Rabbit HRP (7074, cell signaling), diluted at 1:1,000, was employed. Blots were stripped with Restore Western Blot Stripping Buffer (Pierce) and re-probed with monoclonal β-actin (A1978, Sigma) at dilution 1:2000 or polyclonal GAPDH (2118, cell signaling) at dilution 1:5000 as a loading control. H3 (4499, cell signaling) at dilution 1:2,000 and GAPDH (2118, cell signaling) at dilution 1:5,000 are used as loading control for nuclear and cytoplasmic fractions. The western blot was quantified using Image J software version 1.48 (NIH, Bethesda, MD) to determine the band’s density. All the band densities were normalized with loading control β-actin and GAPDH.

**RNA isolation and quantitative RT-PCR**

RNA from liver tissues was extracted using TRIzol Reagent (15596026, Invitrogen) in accordance with the manufacturer instructions (10,13). RNA was transcribed to cDNA using a High-Capacity cDNA Reverse Transcription kit (4368814, Applied Biosystems), as specified by the manufacturer (10,13). Host genes were amplified using SYBR Green (4309155, Applied Biosystems) and specific primer sets (Table S2). All genes were normalized to GAPDH gene expression. Data were represented as fold changes in gene expression using the 2^-ΔΔCt^ method, and each reaction was performed in triplicate.

**Bacterial burden determination**

The number of live replicating intracellular bacteria *Ehrlichia* (IOE or EM) in infected HCs and infected mice was determined using 16s rDNA primers using the 2^-ΔΔCt^ method as previously described. Additionally, the conserved *Ehrlichia* *dsb* gene was amplified using primers (Table S2) to determine the absolute number of *Ehrlichia*.

**Mitochondria isolation, cardiolipin staining, Mitochondrial membrane potential assay.**

Mitochondria was isolated from HCs using mitochondrial isolation buffer following manufacture recommendations (Biovision). For analysis of oxidized cardiolipin, isolated mitochondria were stained by an annexin-V antibody (Biolegend) and analyzed by BD-LSR II (BD, Sans Jose, CA) flow cytometry. The mitochondrial membrane potential was measured using the JC-1 kit (Biotium). In brief, HCs were incubated with JC-1 staining solution for 30 minutes at 37°C and then processed for analysis. The samples were analyzed using BD-LSR II (BD, San Jose, CA) flow cytometry. In both cases, data were analyzed using FlowJo software (TressStar, Ashland, OR)

**DCFDA / H2DCFDA Cellular ROS Assay**

HCs were grown on coverslips and subjected to D3T treatment or infection or both, as indicated by experimental conditions. Cells were stained with DCFDA solution using the DCFDA / H2DCFDA assay (Abcam). Biotek Cytation 5 is used to measure the fluorescent and results were presented as fold change.

**Cell Viability Assay**

HCs were cultured and maintained according to the previously described protocol. Infection was set up using IOE and EM bacteria at an MOI of 1:10. The HCs were treated with varying doses of D3T, followed by the infecting with IOE after two hours. All cells were collected after 24 hours for cell viability. HCs were cultured under different conditions to determine cell viability, and the MTT kit (Abcam) was used to measure cell viability following the manufacturer's instructions.

**IncuCyte Cell Proliferation Assay.**

HCs were cultured in 12-well plates and conditions were set up as dictated by experimental design (Fig. S1E). Continuous monitoring of the cells was conducted for 68 hours. All images were acquired and analyzed to determine cell proliferation. The IncuCyte system employs AI-based technology for image analysis. A detailed protocol can be accessed through the IncuCyte website (https://www.essenbioscience.com/en/protocols/incucyte-cell-count-proliferation-assay-general-pr/).

**Confocal microscopy**

HCs were grown on coverslips and subjected to D3T treatment or infection or both, as indicated by experimental conditions. HCs were grown on coverslips and subjected to D3T treatment or infection or both, as indicated by experimental conditions. To analyze mitochondrial viability or damage, cells were stained with Mitotracker Red (Life Technologies), stained with DAPI, and assessed with scanning laser confocal microscope (Zeiss LSM 710 META). Image Analysis was performed using DP software compatible with Zeiss LSM Confocal Microscope (10,13).

**Statistical Analysis**

The two-tailed t-test was used to compare mean values for two experiment groups. All the data were compiled from at least three different trials that had similar outcomes. All statistical analyses were performed using Graph Pad Prism (GraphPad Software Inc, LA Jolla, CA, USA). Standard deviation and average were used to represent the data. Differences were deemed to be marginally (*), moderately (**), and extremely (***) significant, respectively, with p values of < 0.05, <0.01, and <0.001.
