# Supplementary material for: Evasion of host antioxidative response via disruption of NRF2 signaling in fatal Ehrlichia-induced liver injury
Source: PLoS Pathog. 2023 Nov 13;19(11):e1011791. doi: 10.1371/journal.ppat.1011791 (PMC10681308; doi:10.1371/journal.ppat.1011791)
Supplement: Attachment — Submitted filename: Response to Reviewers.docx [file ppat.1011791.s005.docx]

Dear Editor,

We thank the reviewers for their promising feedback on our manuscript. Their insightful suggestions for the improvement of the paper have been taken into account, and we have performed additional experiments within the given timeline for revision. The details of these experiments will be provided in our point-by-point rebuttal. We have also incorporated additional data into the revised manuscript, which has improved the overall quality of the manuscript. We sincerely appreciate the valuable input from the reviewers, which has greatly aided in this process. Furthermore, we have made several textual modifications highlighted in the revised manuscript and explained in the point-by-point responses.

**Response to Reviewer:**

**Reviewer 1:** The strengths of this manuscript include a well-controlled set of studies demonstrating the role of NRF2 in the liver injury and sepsis mediated by Ehrlichia. Several in vivo and in vitro studies have been used to validate the findings. While much of the transcriptional data is well controlled, there are some issues with the localization of NRF2 to nucleus/cytoplasm based on the immunoblots included. A cleanup of this issue will aid in clearly defining the role of NRF2 in ehrlichial pathogenesis. Histopathology slides are difficult to interpret unless there is guidance on what to look for. Too many gene acronyms are used across the manuscript. A table indicating the list, their role and up/down regulation in uninfected, EM or IOE cells will aid in the understanding of this manuscript.

**Response:** We thank the reviewer for their valuable support of our manuscript. We have addressed the reviewer's comments in our rebuttal letter and included the changes in the manuscript. Please find the point-by-point response in the rebuttal letter below.

**Reviewer 1**

**Point #1:**

Numerous genes -both chromosomal and mitochondrial- are described in this manuscript and is extremely difficult to follow due to differential regulation of these genes and extensive use of acronyms. It is critical to simplify by including a table with genes/proteins analyzed between IOE vs EM vs uninfected cellular/host responses in a table. The authors can use relative indicators (+, ++, +++ or -, --, ---) with gene identities, their role in autophagy/oxidative stress response etc. to simplify the data presented.

**Response:**

We thank the reviewer for suggesting a summary table. We included Table S1 in the revised manuscript, which summarizes all the genes described in this study, their functions, roles in different cellular processes, and changes in protein and/or RNA levels observed among different experimental conditions or mice groups. Table S1 is included in the supplementary data and a table description was added to the discussion page (page 17, line 344).

**Point # 2:**

The summary figure (8C) is also difficult to follow as many of the acronyms used in the figure are not expanded in the figure legend.

**Response:**

We have included the full form of acronyms used in Figure 8C in the figure legends (highlighted).

**Point # 3**

Figure 1. There is no clear description of this figure in the legend or in the description of the results. A-C is combined to state multiple inflammatory infiltrates, necrotic and apoptotic cells steatosis on day 7. Where can this be distinguished in IOE infected sections versus uninfected or EM infected sections? Insets to distinguish these histological changes need to be marked or addressed individually in the results section. The reviewer was unbale to distinguish significant differences other than in 1B and it is unclear what that is supposed to reveal. More definition is needed both in the results section and in the figure legends and descriptions are very general and include multiple statements with no clear representations. How is steatosis distinguished between IOE and EM.

**Response:**

We have rewritten the figure legends for Fig 1 (highlighted). We have introduced two new figures (Fig 1C and 1D) to demonstrate clearly the different pathologic findings in the H&E sections of the liver tissues harvested from lethally/IOE infected mice on day 7 post infection, namely necrosis, steatosis, and hepatic inflammation. The accumulation of fat droplets in the liver cells indicates a potential disruption in lipid metabolism, which can contribute to liver damage and dysfunction We have indicated these pathologic changes at sections with 40x magnification by multiple black arrows. For analysis of apoptotic cells, we had the TUNEL staining presented in Fig 1E and the quantification of apoptotic hepatocytes in Fig 1F.

**Point #4:**

Figure 2A: There are two bands in the immunoblots with anti NRF2. Looks like the lower band is NFR2. If so, an arrow indicating that should be used. Is the upper band a cross reactive band or is it a different version of NFR2?.

**Response**:

The NRF2 migrates between 95–110 kilodaltons (kDa) as described (29). The antibodies that we used in the manuscript were a rabbit anti-mouse NRF2 polyclonal antibody (A1244- abclonal), which recognizes NRF2 protein between 95-100 kDa, which account for the two bands we have seen. However, the lower band corresponds to NRF2 at 95 kDa. We have repeated the *in vivo* experiment described in Fig 2 along with western blot and used freshly prepared antibody as described in Material & Methods. The new NRF immunoblot data showed only one band at 95 kDa, which was less expressed in the liver lysates from IOE-infected mice compared to uninfected and EM-infected WT mice. The new WB has been added to the revised manuscript as Fig 2A.

**Point # 5:**

There is a major discrepancy in the levels of TXNRD1 levels being significantly elevated with IOE infection. There is no discussion of this deviation and in line 288 in the discussion section TXNRD1 is indicated as downregulated. This aspect of Fig 2 needs to be clarified and levels of TXNRD1 should be discussed in the context of this discrepancy.

**Response**:

We thank the reviewer for highlighting the lack of discussion related to the unexpected and discrepant high levels of TXNRD1 compared to other NRF2-transcribed genes and the unintentional error in the discussion. Indeed, our data consistently demonstrated an increased expression of TXNRD1 during fatal IOE infection. As we indicated in the new Table S1, TXNRD1 belongs to the family of thioredoxin reductases, which detoxify bacterial toxins, electrophilic compounds, environmental toxins, and reactive intermediates. TXNRD1 is also regulated by transcription factors other than NRF2 (55). Fatal ehrlichiosis in mice and humans results in significant liver damage associated with a life-threatening toxic-shock-like syndrome, which is characterized by the development of high bacterial burden in liver, systemic cytokine and chemokine storm, dysregulated and excessive hepatic inflammation and cell death followed by liver failure and then multi-organ failure. Thus, the substantial upregulation of TXNRD1 in liver tissues of IOE-infected mice occurs as a counter-protective detoxification host response to ameliorate tissue damage and high bacterial burden. The finding that TXNRD1 is highly upregulated while other NRF2 downstream gene targets such as GPX4 and NQO1 are downregulated suggest that TXNRD1 expression is partially dependent on transcriptional activation signal by NRF2 and other signaling pathways such as USF2 mediated upregulation of TXNRD1 may play a role in this disease process (20,55). This analysis and discussion are currently added in the revised manuscript on page 17, line 355.

**Point # 6**:

Fig 2D. There are two bands detected with anti NRF2 antibodies. In the nuclear fractions, the top band is downregulated. Is this NRF2 translocated to the nucleus? If so, it should be indicated by two arrows, one for nuclear and the other cytoplasmic. There seems to be some inconsistencies in the levels of nuclear and cytoplasmic levels in various immunoblots. The authors can remove this discrepancy by indicating the nuclear and cytoplasmic versions and quantify each version in all immunoblots. Is the relative quantitation done for each band or for both bands together? The identity of the bands used for quantitation should be indicated in the figure legend and arrows for nuclear and cytoplasmic versions included in each immunoblot.

**Response**:

We apologize for the lack of clarification of the molecular weight of NRF2 on multiple WB as well as lack of clarifications of subcellular localization of NRF2. As indicated in our response to point # 4, the NRF2 migrates between 95–110 kilodaltons (kDa) as described (29). The antibodies that we used in the manuscript were a rabbit anti-mouse NRF2 polyclonal antibody (A1244- abclonal), which results in two bands in some cell types as described by manufacture. However, the correct MW of NRF2 within the two bands is 95 kDa.

As suggested by the reviewer, we have examined the NRF2 levels in the nuclear and cytoplasmic fractions of liver lysates using freshly prepared antibodies as described in detail in the Material & Method. We have examined the cytosolic and nuclear fractions on the same blot using appropriate loading controls H3 and GAPDH for nuclear and cytoplasmic fractions, respectively, to exclude any possibility of cross contamination (Fig 2D). We have loaded the total lysates, nuclear and cytoplasmic fractions on the same membrane. We also provided quantification of the nuclear and cytosolic fractions of NRF2 in different conditions in Fig 2E. The identity of all bands used for quantitation are now indicated in the figure legend and we placed arrows when indicated. We added the following paragraph explaining the results on page 8 line 157 “Notably, we found that infection with IOE resulted in a significant decrease in the nuclear NRF2 in liver lysates compared to naive mice (Fig 2D and 2E), suggesting an impaired NRF2 nuclear translocation, expression and activation. Additionally, NRF2 level in the cytoplasmic fraction of IOE-infected liver cells was significantly decreased compared to uninfected mice. Since NRF2 is known to be constitutively degraded prior to its translocation to nucleus and activation, our data suggest that lack of cytosolic expression of NRF2 is likely due to proteasomal degradation. The nuclear translocation and expression of NRF2 and activation in uninfected mice was associated with expression of both nuclear and cytosolic GPX4 isoforms. In contrast, we only detected nuclear, but not the cytosolic, GPX in IOE-infected liver lysates (Fig 2D and 2E). In mice and humans, distinct GPX4 isoforms with different subcellular localization are known to be produced through alternative splicing and transcription initiation; cytosolic GPX4, mitochondrial GPX4 (mGPX4), and nuclear GPX4 (nGPX4) (30). The cytosolic GPX4 is the isoform essential for cell survival and prevention of lipid peroxidation and ferroptosis cell death (31, 32). Thus, these data suggest that defective nuclear translocation and expression of NRF2 and activation is associated with significant downregulation of cytosolic GPX4.

**Point # 7:**
Fig 3. The small molecule D3T used in the study has been used extensively in several studies. There are no relevant references (Line 146) to indicate that this small molecule mediates its function via NRF2. It is important to include a few refs as it appears that this small molecule is being validated for the first time even if it is true for study of oxidative stress response in murine ehrlichiosis.

**Response:**

We have provided relevant literature for small molecule D3T, which is used in our manuscript. We have also added references, which demonstrate that D3T mediates NRF2 expression that induces protective antioxidant response (Reference 33, 34) page 9, line 184.

**Point # 8:**

Fig 3A: is there a difference in nuclear and cytoplasmic levels of NRF2 in the presence of D3T?

**Response:**

Yes. There is a difference based on the normalization of protein expression in the cytosolic or nuclear fractions to the appropriate loading control, GAPDH and H3, respectively as suggested by the reviewers.  Consistent with *in vivo* data, IOE infection resulted in significant decrease in activation of NRF2 as evidenced by substantial lack of NRF2 expression in the nuclear fraction of HCs when compared to uninfected cells (p<0.001) (Fig 3B). Notably, we did not detect NRF2 expression in the cytoplasmic fraction of IOE-infected cells, suggesting that cytosolic NRF2 may undergo proteasomal degradation at that time point. Importantly, treatment of IOE-infected HCs with D3T resulted in significant NRF2 nuclear translocation (p<0.001) as marked by increased expression of nuclear NRF2 compared to infected but untreated cells (Fig 3B).

The new data are now added as Fig 3B and in the result section on page 10 line 188.

**Point # 9:**

How is the discrepancy between *in vitro* and *in vivo* data with regards to TXDR4 resolved? Which is more relevant? There needs to be some discussion about this.

**Response:**

We thank the reviewer for raising this important point. Our data in Fig 2C indeed shows a discrepancy in the levels of TXNRD1 being significantly elevated *in vivo*, in the liver of IOE-infected mice, while other antioxidants such as NQO1 and GPX4 are decreased. We provided an explanation for this discrepant data in our response to point # 5. However, consistent with the *in vivo* findings, the expression of TXNRD1 *in vitro* in IOE-infected HCs was increased compared to uninfected cells, and that increase was further enhanced upon treatment with D3T (Fig 3D).

**Point # 10:**

Fig 6B. The distortion/disorganization of ER appears to be the same between EM and IOE infected cells compared to uninfected cells. The authors may want to show another image or reword this sentence (Line 233). ER in uninfected cells is very clear while it appears to be the
same in Em/IOE infected cells. References needed after line 237/238.

**Response:**

We agree with the reviewer. TEM analysis showed that infection of liver with EM or IOE leads to disorganization or distortion of ER compared to uninfected liver. However, mitochondrial cristae and structure was intact in EM-infected liver tissues. On the other hand, IOE-infected liver exhibited dysfunctional mitochondrial with abnormal mitochondrial morphology and disorganization with matrix swelling and collapsed cristae, suggesting mitochondrial dysfunction (Fig 6B). We have included the description of this data on page 14 line 281. We have included references 49 and 50 on page 14, line 291.

**Point # 11**

Fig 7E. Is there a statistical difference in weight of liver between D3T treated and D3T treated plus IOE?

**Response:**

There is no statistical difference in weight of liver between D3T treated and D3T treated plus IOE. We revised this section as follows to clearly analyze the significant differences between multiple groups of mice: Treatment of uninfected mice with D3T did not significantly influence liver weight when compared to uninfected and untreated mice (Fig 7E). Infection of mice with IOE significantly (p< 0.05) increased liver weight compared to uninfected mice (Fig 7E). However, attenuated mortality in D3T treated, IOE-infected mice were associated with significant decrease in the weight of the livers and spleens, decreased total number of splenocytes, as well as decreased bacterial burden when compared to untreated IOE infected mice (Fig 7C and 7E) (Fig S1F). Notably, the weight of IOE-infected liver was significantly decreased with D3T treatment, suggesting that restoration of anti-oxidative response prevent hepatomegaly induced upon fatal IOE infection. Page 15, line 303.

**Point # 12:**

Fig 8. Histological changes reflecting the pathology need to be marked/focused on the sections as it appears similar in all three sections to a reader.

**Response**:

We have rewritten the figure legends for Fig 8 (highlighted). Also, we have indicated inflammatory infiltrates cells with blue arrows, steatosis using white arrows and necrosis with yellow arrow (Fig 8A). We observed increased steatosis in the IOE-infected liver tissue in the histological images only and there is a marked decrease in steatosis, infiltrates inflammatory cells and necrosis upon D3T treatment. (Fig 8A). In Fig 8B, we have indicated apoptotic Kupffer cells and hepatocytes using arrows and arrowhead respectively. We have quantified the average number of apoptotic cell count of kupffer cells and hepatocytes in indicated groups (Fig S1G).

**Point # 13:**

A table listing all the chromosomal and mitochondrial genes/their functions with their noted effects will aid in a greater understanding of the proposed studies.

**Response:**

We have included a table having the list of all genes and their function as mentioned. Please find Table S1 in the supplementary data.

**Reviewer 2:**

**Reviewer 2**:  The important finding of this study is that the virulent strain of Ehrlichia (IOE) inhibits the expression and nuclear translocation of the NRF2 transcription factor in hepatocytes. NRF2 acts as a master regulator of redox homeostasis and metabolic balance during infection, protecting the host against oxidative stress by upregulating essential antioxidants. Thus, the inhibition of NRF2 expression and activity by IOE is proposed as a mechanism underlying severe inflammation and liver injury in ehrlichiosis….. Overall, the study presents novelty and is of broad interest to researchers working in the field of pathogen-host interactions.

**Response:**

We truly appreciate the reviewer’s positive comments about our study. We have provided all the clarification with results, approaches, and method choices as discussed in our point-by-point reply.

**Point # 1:
Figure 2D**: Nuclear translocation data are not supported by appropriate controls. Additional WB required to control for the purity of the fractions – addressing expression of nuclear protein (lamin, histone 3, PARP, any other) and cytoplasmic protein (GAPDH, Beta-tubulin, etc) on both fractions on one membrane for direct comparison of fractions. Ideally, total lysates should be loaded on the same membrane as well. Loading control is important, but it is not providing control for the proper fraction’s separation – possible contamination of cytosolic fraction with ruptured nuclear content or possible contamination of nuclear fraction by cytosolic proteins if nuclei were not properly washed before lysis.

**Response**:

We have addressed the concerns of the reviewers in the manuscript. We have repeated the experiments in new Figures 2D and 3B with nuclear control (H3 protein) and cytoplasmic control (GAPDH protein) and we included correct normalization of the protein expression based on the loading controls for each fraction.

**Point # 2:**

APPROPRIATE SECTION describing the method should be added to the materials and methods part of the manuscript – how the separation was performed???

**Response:**

We apologize for missing important details related to nuclear and cytoplasmic fractionation. We have provided the following methodological details in the supplemental data.

“For *in vivo* data, 20 mg of the liver tissue was cut, washed with PBS, and dried. The liver tissue was then resuspended into 200 ul of ice-cold CER-I buffer and homogenized using a Dounce homogenizer. For *in vitro* culture, HCs were harvested and centrifuged at 500 g for 5 minutes to collect cells. For isolation of the nuclear and cytoplasmic proteins from liver lysates or HCs, we used NE-PER™ Nuclear and Cytoplasmic Extraction kits as recommended by the manufacturer (Thermofisher). All steps were performed on ice. Briefly, cells were washed with PBS and centrifuged at 500 × g for 5 minutes to obtain pellets. The supernatant was discarded, and the cell pellet was resuspended in 100 µl of ice-cold CER-I. The pellet was vigorously vortexed and then incubated in the CER-I buffer for 10 minutes. Following this incubation, ice-cold CER-II was added to the samples, vortexed, incubated for 1 min, and centrifugation at 16,000 x g for 5 minutes. The cytoplasmic supernatant was collected to clean pre-chilled tube on ice. The pellet containing nuclei was resuspended in NER-I reagent, and the resuspended pellet was vortexed for 15 sec every 10 minutes for a total of 40 minutes. The nuclear fraction was then centrifuged at 16,000 x g for 10 minutes and the supernatant containing the nuclear extract was transferred to a clean pre-chilled tube. The cytosolic and nuclear extracts were stored at -80°C until needed. Approximately 10 ug of the cytosolic and nuclear extracts were subjected to analysis through 4-20% SDS-PAGE and subsequent Western blotting. The primary antibodies targeting specific proteins were appropriately diluted at 1:1000 for NRF2, 1:2000 for β-actin,1:2,000 for H3 and 1:5,000 for GAPDH. The secondary antibody, anti-Rabbit HRP, diluted at 1:1000, was used”.

**Point # 3:**

Could you comment on GPX4 size (below 17 kDa) as well as its function in the nucleus? Is it 22 kDa or shorter?

**Response:**

We have corrected the labeling for GPX4 molecular weight. The observed molecular weight of GPX4 is 21 kDa.

As for the function of nuclear GPX4, the extent of research into the function of nuclear GPX4 is limited. However, in mice, distinct GPX4 isoforms with different subcellular localization are known to be produced through alternative splicing and transcription initiation; cytosolic GPX4, mitochondrial GPX4 (mGPX4), and nuclear GPX4 (nGPX4). The cytosolic GPX4 is isoform essential for cell survival and prevention of lipid peroxidation and ferroptosis cell death (31,32). In humans, experimental evidence for alternative splicing exists with cytoplasmic GPX4 being the key isotype required for protection against oxidative stress. However, nuclear GPX4 isoforms (phospholipid-hydroperoxide glutathione peroxidase, PHGPx) has been implicated in spermatogenesis and male fertility (30). We included this information in the result section on page 9 line 167.

**Point # 4:**

Do you have the total lysates to complement your mRNA expression data for GPX4 from Figure 2C by total protein expression level using WB on total lysate? (Like it as was done for total NRF2 on Figure 2A)?

**Response:** We have included the western blot of GPX4 in new Figure 2B, showing the differential expression of GPX4 in Uninfected, EM-infected, and IOE-infected liver tissue. We also added these results in the text on page 8 line 147 and figure legend.

**Point # 5:**

Figure 7: It is a mess with subfigures description in the legend – should be checked and corrected by authors according to the presentation on the figure. Figure 1D, Figure 7B should have statistical significance and number of animals per group on the figure and figure legends.
**Response:**

We understand the concern raised by the reviewer regarding the mismatch in the presentation of data in Figure 7 and its figure legends. We have corrected this mismatch. We have also included statistical significance and the number of animals/groups in Figure 1D & Figure 7B and their figure legends.

**Point #6:**

There are several repetitions of same conclusions that should be corrected, text requires English editing (example – lines 297-299; lines 310-312; lines 339-341)

**Response:**

We have edited the manuscript for grammar and English and deleted redundant sections as suggested. Please refer to page 18, line 377; page 19, line 388; page 20 line 419.

**Point # 7:**

References for statements required in lines 271-274. Line 277 – activation of caspase-11 is downstream inflammasome activation, not vice versa, like it reads now. Line – 288 – please list some processes and provide references.

**Response:**

We have provided references on page17, line 335 (reference-51,52); page 17, line 338 (reference-6,7); page14, lines 291 (reference-49,50).

We have changed “caspase 11 mediated inflammasome pathway” to “non canonical inflammasome pathway marked by caspase 11 activation” (Page 17 and 341).

We have provided references on page17, line355 (reference-21,22,25,28).

**Point #8:**

Statement on lines 317-318 requires reference. Could you comment if the mentioned strain (line 317) is virulent, how it is related to the strains that were used in current study.

**Response:** We have included the required reference 59-61 (page19 and line 395).

The *Ehrlichia chaffeensis* strain is a virulent *Ehrlichia* species. It is the main etiology of Human Monocytic Ehrlichiosis (HME). In humans, different strains of *Ehrlichia chaffeensis* exhibit distinct virulence traits, each delineated by specific genomic sequences (64). However, this species causes abortive infection in immunocompetent mice and thus it is not ideal model to use for analysis of immunopathogenesis of fatal ehrlichiosis. Alternatively, the two strains used in this study EM and IOE are closely related to *E. chaffeesnis* (63, 64). Infection of mice with EM and IOE causes nonfatal and fatal ehrlichiosis, respectively that recapitulate clinical, laboratory, immunological and pathological findings in patients with mild and fatal ehrlichiosis (7, 8, 65). Therefore, these infection models are ideal to dissect the molecular and cellular mechanisms that account for the development of mild or severe and potentially fatal HME. We have included this detail in the manuscript on page19 line 399.

**Point #9:**

**4)** Line 329 – make sure that the journal allows to refer to unpublished data, usually it is not accepted. Line 332 – could you suggest if stability or complex with Keap-1 could be also affected.

**Response:**

We have removed the statement from the manuscript (line 410) in which we refer to unpublished data.

Decreased translocation of NRF2 during IOE infection could be explained by stabilization of the KEAP1-NRF2 system. KEAP1 is an adaptor of the ubiquitin ligase complex that targets NRF2 for degradation. NRF2 is an unstable complex that tends to degrade quickly, which may explain the lower expression level of total NRF2 in hepatocytes and livers.

**Point # 10:**

Again – check that all methods were included, like for example the missing section on nuclear/cytosolic fractions isolation.

**Response:**

We have included all the methods and materials in the main manuscript and supplementary file. We have also included all the details for Nuclear/Cytosolic fraction isolation and western blot procedure in the manuscript.

**Point#11:**

Line 361 - Mice sex (male, female), time for acclimation before the experiment? Day-light cycle? There are certain protocols to describe the mouse experiments recommended by the journal as well that should be followed.

**Response:**

Female 7–8-week-old C57BL/6 were used in the experiments and obtained from Jackson Laboratory (Bar Harbor, ME). All the mice were maintained in a specific pathogen-free environment under a consistent light-dark cycle required for regulation and maintenance of the mice’s circadian rhythms and behavior. The experiment conducted in the study was carried out under the Assurance Number 00290(A3460-01) and IACUC protocol number 21-117 approved by the Institutional Animal Care and Use Committees, University of Illinois. Please find these details in the Material and Methods.

**Point # 12**

Line 364 – decipher IOE strain again, provide the source for both strains that were used in the study. Line 366 – for D3T – what type of injection? Section Western blot (line 379) – as to the antibodies you should state if they were produced in mice, or rabbit, or rat, or goat, or etc.

**Response:**

We have deciphered the IOE strain again as *Ixodes ovatus Ehrlichia* (IOE, AKA *E. japonica*) in line 109. We have provided the details of D3T injection (intraperitoneal injection) in the manuscript- line 111 under Material and Methods. We have mentioned the source of antibodies in the Material and Methods (Supplemental data).

**Point # 13**

RNA isolation and quantitative RT-PCR, bacterial burden determination – I request to provide all primers in main text or in supplementary file.

**Response:**

We have included all the primers used in the manuscript in the supplementary file (Table S2).

 **Minor Comments:**

**Point #1:**

All genes’ titles should be correctly marked on graphs (italic, small letters for murine genes) and all throughout the text of the manuscript to separate where the authors address or describe protein expression, and where they examine mRNA expression of certain genes.

**Response:**

We have incorporated all the changes in the manuscript.

**Point #2:**

I would recommend deciphering the abbreviation EM early in the beginning of the results part (line 105)

**Response:** We have incorporated the changes in the manuscript in line 109.

**Point #3:**

Section (after line 218) should contain brief explanation for the choice of genes addressed in the study and abbreviations should be also explained. Why these genes? Any references that could be provided to explain the choice?

**Response:** We have included the complete list of genes with gene categories, identities, abbreviations, functions, and results with relative indicators in new Table S1 (Supplementary data).

**Point #4:**

**4)** Lines 147-248: Is it only about expression or also the stability of NRF2 protein – disconnection from Keap1? Both? Is it possible to address?

**Response:**

NRF2 shows a short half-life of approximately 20 min in the cell; however, it is extended upon disruption of NRF2-KEAP-1 interaction. Once the interaction between NRF2 and its potent repressor, KEAP-1, is disrupted, NRF-2 translocate to the nucleus to increase expression and its downstream genes. We have examined the NRF2 levels in nuclear and cytoplasmic fractions (hepatocytes) (Fig 3B). Our results demonstrated that uninfected hepatocytes have more nuclear NRF2 than IOE-infected hepatocytes. It indicates impaired translocation, expression, and activation of NRF2 in IOE-infected hepatocytes. This decrease in NRF2 upon IOE infection can be restored using D3T (NRF-2 inducer). This indicates that NRF2 translocation, expression, and activation play an essential role in IOE pathogenesis.

**Point #5**

Figure 3B-D – what was the concentration of D3T selected for these assays – please note in the figure or figure legend. Remove mark (µm) from Figure 4C, D or write the concentration used - 2.5 µM.

**Response:**

We have addressed these comments in the manuscript. We have removed the extra µm mark and provided D3T concentration – 2.5 µm in the figure’s legends.

**Point #6**

Have you tried staining Nrf2 in liver sections of infected mice? Is it possible to compare the expression level visually, by comparing the staining intensity in levers of uninfected, EM- and IOE-infected mice by fluorescent staining of sections for example?

**Response:**

Unfortunately, due to limited time of responding to the reviewers; comments, we did not perform fluorescent staining of liver sections. However, our prior and new western blots showing NRF2 protein expression in nucleus and cytoplasm fraction in uninfected, infected and D3T treated samples as well as the complementary *in vitro* and *in vivo* approaches strongly and consistently suggest decreased nuclear translocation of NRF2 and thus defective activation of downstream targets.

**Point #7**

Figure 5C, 6D – molecular size should be noted on WBs.

**Response:**

We have included the molecular weight in the figures.

**Point #8**

Is it correct to state “NRF2 signaling pathways” (line 198, line 283)? Because NRF2 is not activating directly the signaling events, but rather activates certain gene expression profile as a transcription factor.

**Response:**

We have addressed these changes by modification of the sentence. “Activation of NRF2 signaling pathways changed to Increased NRF2 expression in line 337. NRF2 activation is changed to “NRF2 expression.” in line 339.

**Point # 9**

Line 429 – not clear, should be edited – “treated to D3T treatment”? Line 431 – specify “a large number” by providing the actual range for the number.

**Response:**

Corrected. Please see the changes in the Material and Method section in the supplementary file.
