# Supplementary material for: Evasion of host antioxidative response via disruption of NRF2 signaling in fatal Ehrlichia-induced liver injury
Source: PLoS Pathog. 2023 Nov 13;19(11):e1011791. doi: 10.1371/journal.ppat.1011791 (PMC10681308; doi:10.1371/journal.ppat.1011791)
Supplement: Attachment — Submitted filename: Response to Reviewers.docx [file ppat.1011791.s006.docx]

Dear Editor,

We sincerely appreciate the thoughtful and constructive feedback from the reviewers, which has helped us to improve our manuscript. We have made textual modifications and highlighted them in the revised manuscript. Please see the point-by-point responses for more details below.

**Response to Reviewer:**

**Point #1:**

I would highly suggest to change the color of arrows in Figure 1A, B - black arrows are almost not visible in printed text
**Response:**

We have changed the color of the arrows to a more visible color Yellow in Fig1A, B. For Fig1E, we have increased the size of the arrows and changed the color to Blue for better visibility. We introduced similar changes in Figure 8A, B.

**Point #2:**

Figure 2 - mention the meaning of T, N, C abbreviations in the brackets in the figure legend - same for Figure 3 B

**Response:**

We have updated the figure legend for Figure 2D and 3B, with the abbreviation T as Total Protein Lysate, N as Nuclear Protein Lysate and C as Cytoplasmic Protein Lysate.

**Point #3:**

Figure 3 title - "increase in NRF2" - expression? activity? stability? - be specific
**Response:**

We have revised the title in the figure legends.

**Point #4:**

Figure 5, 6 and 7 - it would be important to add molecular weight values for the blots presented on these images as you have done for the blots in other Figures. You have mentioned in the answer to the reviewer that it was done, but it was not done in reality.

**Response:** We apologize for this oversight and thank the reviewer for pointing this out. We have listed now the molecular weights of proteins detected in each western blot in the manuscript.

**Point #5:**

Figure 6D - you should mark in the figure legend if the protein samples were run in the same gel and quantified from the same membrane
**Response:**

Thank you for your suggestion. Indeed, the protein samples from uninfected and infected cells, with or without TUDCA inhibitor was run in the same gel and data are the density of bands was quantified from the same membrane. We have included this sentence in the figure legend of Fig. 6D.

**Point #6:**

Text and figures - use uniformly the term cytoplasmic fraction - you use both cytosolic and cytoplasmic terms in the text and in the figures and legends.
**Response:**

We have included the changes into the manuscript and now used “cytoplasmic fraction” consistently (changes highlighted in the manuscript).

**Point #7:**

Line 162 - There is a significant decrease of NRF2 expression in total lysates along with both nuclear and cytoplasmic fractions (Figure 2D). It is showing the effect of IOE on the NRF2 stability at the first place. In line 163 you rather put forward translocation and expression, however, there are no mRNA expression data for Nrf2 gene in this figure. This part of the text and conclusions should be edited.

**Response:**

We have updated the text to reflect our findings on page 9 and 10, lines 163-170 as well as in the conclusion on lines 177-179.

**Point #8:**

Line 310 - edit the text, and it is a general comment for all newly inserted sections
**Response:**

We have revised the text in line 312 to ensure that the conclusion for the results is more precise and accurate.

**Point #9:**

Line 318-319 - I would suggest to change to: In contrast, treatment of IOE infected mice with D3T not only decreased the activation of caspase-11 in liver tissue ... - isn't it more correct?

**Response:**

Yes. That is correct. We changed the sentence accordingly to state that” In contrast, treatment of D3T to liver tissue not only decreased activation of caspase 11, but also decreased protein expression of pro-caspase 11, suggesting that IOE-mediated, NRF2-dependent oxidative stress promote caspase 11 activation as well as regulate pro-caspase 11 expression.”

**Point #10:**

Discussion - I would suggest moving and combining new text from line 345-347 to the line 356

**Response:**

We have moved the text from line 345 to line 356 and combined the sentences in the discussion section.
